# An Assessment of the Novel COVISTRESS Questionnaire: COVID-19 Impact on Physical Activity, Sedentary Action and Psychological Emotion

**DOI:** 10.3390/jcm9103352

**Published:** 2020-10-19

**Authors:** Ukadike Chris Ugbolue, Martine Duclos, Constanta Urzeala, Mickael Berthon, Keri Kulik, Aura Bota, David Thivel, Reza Bagheri, Yaodong Gu, Julien S. Baker, Nicolas Andant, Bruno Pereira, Karine Rouffiac, Maëlys Clinchamps, Frédéric Dutheil, on behalf of the COVISTRESS Network

**Affiliations:** 1 Faculty of Sports Science, Ningbo University, Ningbo 315211, China; guyaodong@hotmail.com (Y.G.); jsbaker@hkbu.edu.hk (J.S.B.); 2 Institute for Clinical Exercise & Health Science, School of Health and Life Sciences, University of the West of Scotland, South Lanarkshire G72 0LH, Scotland, UK; 3 Department of Biomedical Engineering, University of Strathclyde, Glasgow G11XQ, UK; 4 Department of Sport Medicine and Functional Exploration, University Hospital CHU G. Montpied, INRA, UNH, CRNH Auvergne, Clermont Auvergne University, Clermont-Ferrand, F-63000 Clermont-Ferrand, France; mduclos@chu-clermontferrand.fr; 5 Sports and Motor Performance Department, Faculty of Physical Education and Sports, National University of Physical Education and Sports, 060057 Bucharest, Romania; constanta.urzeala@unefs.ro; 6 LaPSCo, Catech, Université Clermont Auvergne, CNRS, F-63000 Clermont-Ferrand, France; mickael.berthon@uca.fr; 7 Health and Physical Education Program, Indiana University of Pennsylvania, Bloomington, IN 47405-1006, USA; kskulik@iup.edu; 8 Department of Teaching Staff Training, Faculty of Physical Education and Sports, National University of Physical Education and Sports, 060057 Bucharest, Romania; aurabota@ymail.com; 9 Laboratory of Metabolic Adaptations to Exercise under Physiological and Pathological conditions (AME2P), Université Clermont Auvergne, CRNH Auvergne, F-63000 Clermont-Ferrand, France; david.thivel@uca.fr; 10 Department of Exercise Physiology, University of Isfahan, 8174673441 Isfahan, Iran; will.fivb@yahoo.com; 11 Department of Sport, Physical Education and Health, Centre for Health and Exercise Science Research, Hong Kong Baptist University, Kowloon Tong 999077, Hong Kong, China; 12 Biostatistics Unit, DRCI, University Hospital of Clermont-Ferrand, CHU Clermont-Ferrand, F-63000 Clermont-Ferrand, France; nandant@chu-clermontferrand.fr (N.A.); bpereira@chu-clermontferrand.fr (B.P.); 13 Preventive and Occupational Medicine, University Hospital of Clermont-Ferrand, CHU Clermont-Ferrand, F-63000 Clermont-Ferrand, France; krouffiac@chu-clermontferrand.fr; 14 Physiological and Psychosocial Stress, LaPSCo, CNRS, Preventive and Occupational Medicine, WittyFit, University Hospital of Clermont-Ferrand, CHU Clermont-Ferrand, Université Clermont Auvergne, F-63000 Clermont-Ferrand, France; maelysclinchamps@gmail.com (M.C.); frederic.dutheil@uca.fr (F.D.)

**Keywords:** COVID-19, Severe Acute Respiratory Syndrome Coronavirus 2 (SARS-CoV-2), depression, anxiety, stress, distress

## Abstract

Globally the COVID-19 pandemic outbreak has triggered an economic downturn and a rise in unemployment. As a result, global communities have had to face physical, health, psychological and socio-economical related stressors. The purpose of this study was to assess and report the impact of isolation and effect of coronavirus on selected psychological correlates associated with emotions. Following ethical approval, a mixed methods observational study was conducted using the validated COVISTRESS questionnaire. Two observational study scenarios were evaluated namely “Prior” to the COVID-19 outbreak and “Currently”, i.e., during the COVID-19 pandemic. 10,121 participants from 67 countries completed the COVISTRESS questionnaire. From the questionnaire responses only questions that covered the participant’s occupation; sociodemographic details, isolation and impact of coronavirus were selected. Further analyses were performed on output measures that included leisure time, physical activity, sedentary time and emotions. All output measures were evaluated using the Visual Analogue Scale (VAS) with an intensity ranging from 0–100. Descriptive statistics, Wilcoxon signed-rank test and Spearman correlational analysis were applied to the leisure time, physical activity, sedentary time and emotional feeling datasets; *p* = 0.05 was set as the significance level. Both males and females displayed similar output measures. The Wilcoxon signed rank test showed significant differences with respect to “Prior” COVID-19 and “Currently” for sedentary activity (Z = −40.462, *p* < 0.001), physical activity (Z = −30.751, *p* < 0.001) and all other emotional feeling output measures. A moderate correlation between “Prior” COVID-19 and “Currently” was observed among the Males (r = 0.720) in comparison to the Females (r = 0.639) for sedentary activity while weaker correlations (r < 0.253) were observed for physical activity and emotional feeling measurements, respectively. Our study reported incremental differences in the physical and psychological output measures reported, i.e., “Prior” COVID-19 and “Currently”. “Prior” COVID-19 and “Currently” participants increased their sedentary habits by 2.98%, and the level of physical activity reduced by 2.42%, depression levels increased by 21.62%, anxiety levels increased by 16.71%, and stress levels increased by 21.8%. There were no correlations (r) between leisure, physical activity and sedentary action (i.e., “Prior” = −0.071; “Currently” = −0.097); no correlations (r) between leisure physical activity and emotion (i.e., −0.071 > r > 0.081) for “Prior”; and poor correlations (r) between leisure, physical activity and sedentary action (i.e., −0.078 > r > 0.167) for “Current”. The correlations (r) between sedentary action and emotion for “Prior” and “Currently” were (−0.100 > r > 0.075) and (−0.040 > r > 0.041) respectively. The findings presented here indicate that the COVISTRESS project has created awareness in relation to the physical and psychological impact resulting from the COVID-19 pandemic. The findings have also highlighted individual distress caused by COVID-19 and associated health consequences for the global community.

## 1. Introduction

An investigation by the Chinese Centre for Disease Control and Prevention (CCDC) on 7 January 2020 identified a causative agent called Severe Acute Respiratory Syndrome Coronavirus 2 (SARS-CoV-2) from throat swab samples. This disease was classified as COVID-19 by the World Health Organisation (WHO) [1]. COVID-19 infected patients present with dry coughs, sore throats and fever; however, complications occur when these mild symptoms develop into septic shock, pulmonary oedema, organ failure, severe pneumonia and Acute Respiratory Distress Syndrome (ARDS) [2]. Older and obese citizens appear to be more vulnerable if complications develop and intensive care support is required if individual situations worsen due to underlying medical conditions or comorbidities. These comorbidities include cerebrovascular, endocrine, cardiovascular, digestive and respiratory diseases. Cases admitted into intensive care also reported abdominal pain, dyspnoea, dizziness and anorexia [3,4].

A global health emergency based on the COVID-19 outbreak was declared on the 30th of January 2020. It is evident that countries more susceptible to the COVID-19 situation are those with compromised, weak or vulnerable healthcare systems. As the spread of the COVID-19 pandemic continues to grow, efforts continue to be directed towards strategically implementing healthcare guidelines designed to interrupt its course and minimise effects. This means that healthcare systems on a global scale are all engaged in reducing COVID-19 transmission through early detection, isolation, rapid treatment and the execution of an organised and robust system to contact and trace COVID-19 victims and potential carriers of the virus [5]. On the 6th of August 2020, the WHO COVID-19 Situation Report–199 highlighted the fact that on a global scale, the situation statistically (new cases in the last 24 h) had worsened and cases had risen to 18,614,177 (increase of 259,344) with 702,642 deaths (increase of 6488) recorded. This was in addition to new cases being recorded as high and still increasing [6]. In particular, the number of confirmed COVID-19 cases (new cases in the last 24 h) and deaths (new cases in the last 24 h) reported in terms of numbers (by WHO region) on the 6 of August 2020 were as follows: Africa, 848,053 (13,906) cases and 15,252 (502) deaths; Americas, 9,981,204 (139,362) cases and 372,008 (4,074) deaths; Eastern Mediterranean, 1,598,640 (13,182) cases and 42,052 (451) deaths; Europe, 3,477,225 (25,178) cases and 215,168 (430) deaths; South-East Asia, 2,360,721 (61,288) cases and 49,572 (1003) deaths; and Western Pacific, 347,593 (6428) and 8577 (28) deaths [6]. 

In response to the global pandemic, funding for research into the pathophysiology of the virus to the sum of €10,000,000 from the EU has been invested to “contribute to a more efficient clinical management of patients infected with the virus, as well as public health preparedness and response” [7]. A total of £20,000,000 has been invested by the United Kingdom (UK) government into the development of a COVID-19 vaccine [8]. To date one COVID-19 vaccine has been approved whilst others are at the pre-clinical trial phase, Phase 1, Phase 2 and/or making transitions into Phase 3. Gam-COVID-Vac now known as Sputnik V was developed at the Gamaleya Research Institute in Moscow. The Ministry of Health of the Russian Federation approved this vaccine on 11th of August 2020. Despite Russia being the first country to speedily develop a COVID-19 vaccine (Sputnik V), the vaccine is yet to enter Phase 3 Clinical Trials, and experts have raised concerns about the vaccine’s safety and efficacy [9]. In Scotland, the financial impact of COVID-19 has been supported by the Scottish Government with a goal to protect and secure their world-leading research programmes through the provision of a £75 million one-off increase in funding for Scotland’s universities [10]. More recently, various independent and collaborative multidisciplinary related research studies on COVID-19 have been investigated in the area of equipment and manufacturing [11,12,13,14], pathophysiology [15,16,17], neurophysiology [18,19,20,21], radiology [22,23,24,25], psychology [26,27], diagnostics [28,29,30,31,32] and treatment [33,34,35]. 

Despite the funding provisions made available and with research studies initiated globally, the financial impact of COVID-19 among the white-collared and blue-collared workers continues to remain. On a global scale, the COVID-19 pandemic continues to affect overall efficiencies and productivity levels, livelihoods within and outside the health sector, as well as economies, which have resulted in an economic downward spiral. These negative impacts induced by COVID-19 triggered the initiation of this investigation termed COVISTRESS. There are no published findings using the COVISTRESS questionnaire on the physical and psychological impact of the COVID-19 pandemic in the general community nationally or from an international perspective. This mixed methods investigation, i.e., both qualitatively and quantitatively driven, utilises a customised questionnaire focused on studying the impact of the coronavirus on life issues and consequential development of STRESS particularly on individual lifestyle and work environment. The COVISTRESS questionnaire has been validated in-house using similar scientific rigour applied to the published JOBSTRESS* Randomised Trial [36]. The COVISTRESS questionnaire has been translated into nine languages and, to date, distributed in sixty-seven countries. The questionnaire, prior to administration, was translated into the native language of each different country. A native speaker of the country concerned checked the integrity of the translation, to ensure that none of the information regarding the questions was lost during translation. This confirmed that the questions were consistent in every country where the questionnaire was administered.

The questionnaire consists of nine sections requiring the participants in most sections to complete a Visual Analogue Scale (VAS) with an intensity scale ranging from 0–100. Different versions of the VAS have been reported by Dutheil and colleagues [36,37,38]. The nine sections from the COVISTRESS questionnaire cover information relating to epidemiological context, participant’s COVID-19 knowledge, measure of stress and worries, participant’s occupation, VAS and professions, parenthood and family, sociodemographic details, isolation and impact of coronavirus; and participant’s health coverage. Extracted from the COVISTRESS questionnaire are three components namely leisure physical activity, sedentary action and emotions. It is envisaged that an evaluation of these selective components will provide health and wellbeing information and emotions associated with coronavirus stress and its psychological impacts on societies. These three components are important in understanding the influence coronavirus has had on the citizens of the world. Emerging evidence suggest that the Coronavirus pandemic has initiated a devastating threat to our public health, our lifestyles and our countries’ economies. As a consequence of observing quarantine and social distancing measures as well as hospitalization and bed rest measures, physical inactivity brought about by immobilization could downgrade the capacity of organs to remain fully functional and operationally efficient. This means the vulnerability of our organs could potentially become compromised and unable to resist and fight off the likelihood of viral infections. These repercussions could thereby increase the risk of damage to the cardiovascular system, immune system, respiratory system, musculoskeletal system and the brain [39]. Aside from the physiological impact, psychological emotions and metal health factors can also play a role in increasing anxiety levels, loneliness and social isolation during lockdown [39,40,41]. Social isolation (an absence of social connections) and loneliness (subjective dissatisfaction with relationships) although considered as under-recognised determinants of health status [41], both have also been found to be predictors of cardiovascular disease, cognitive decline, increased depression and premature mortality [42,43,44]. The combination of the physiological related (i.e., physical activity, sedentary activity) and psychosocial related (i.e., emotion) variables have not been previously investigated in relation the COVID-19 pandemic.

Therefore, the aims of this study were to assess and report the impact of isolation and effect of coronavirus on leisure physical activity and sedentary action psychological correlates associated with emotional feelings. We hypothesized that the relationship “Prior” COVID-19 and “Currently” will show an increase in physical inactivity and sedentary measurements, and that these outcome measures will instigate definitive emotional feelings connected with anxiety, depression and stress.

## 2. Method

### 2.1. Participants

Following ethical approval from the University hospital in France (Nos Ref.: 2020/CE 06) on the 9 of March 2020, a mixed methods observational study was conducted using the validated COVISTRESS questionnaire. 10,121 participants completed the questionnaire. All participants reviewed the information sheet and voluntarily provided written informed consent prior to participating in the study. Participants were given the freedom to withdraw from the study at any time without having to give reasons. Regarding the research on human participants, the procedures of this observational study complied with the provisions of the Declaration of Helsinki. The inclusion criteria were not restrictive, as both COVID-19 positive and negative participants were recruited onto the study irrespective of whether the participant had contact with a person infected with the virus or not. The principal investigator and co-investigators were interested to ascertain the impact that this pandemic had on the lives of the participants. This study is still active in terms of data collection and is an international collaboration between several Institutions (University Hospitals, Universities, The French National Centre for Scientific Research (French: Centre national de la recherche scientifique, CNRS), and Occupational Health).

### 2.2. Output Measurements 

Participants were recruited via the application of the convenience and snowball sampling technique. During participant selection, all participants that satisfied the recruitment criteria were granted online access to the COVISTRESS questionnaire. The duration of the administration of the COVISTRESS questionnaire was 73 days (from 30th of March 2020 to 10th of June 2020). The participant selection, recruitment and advertisement were achieved through radio, television, fliers, posters, newspapers and online mediums including social networks such as Facebook, Twitter, etc. The anonymous online COVISTRESS questionnaire was completed with data including the participants’ sociodemographic information, health and wellbeing information and psychosocial information. Quality assurance was maintained by ensuring only one questionnaire was submitted per IP address. A master spreadsheet was prepared using Microsoft Excel 2019 version 16.23 (Microsoft Corporation, Redmond, WA, USA) for data processing and analyses. Two observational study scenarios were evaluated namely “Prior” to COVID-19 pandemic and “Currently”, i.e., during the COVID-19 period. From forty-five questions spread across the nine sections, only questions that covered the participant’s occupation; sociodemographic details; and isolation and impact of Coronavirus were selected to analyse the three components, namely, leisure physical activity, sedentary action and emotions. According to the World Health Organisation [45], physical activity is defined as “any bodily movement produced by skeletal muscles that require energy expenditure. Popular ways to be active are through walking, cycling, sports and recreation, and can be done at any level of skill and for enjoyment”. In line with the Public consultation on the draft WHO Guidelines on physical activity and sedentary behaviour for children and adolescents, adults and older adults 2020, sedentary action was defined as any very low energy expenditure activity that requires sitting or lying as the dominant posture [46]. All these components were evaluated using the VAS with an intensity scale ranging from 0–100. In general, VAS is a psychometric response scale that can be used in questionnaires to assess occupational stress [47,48].

In terms of the three components, the information requested was the following: Number of hours of leisure physical activity per week “Prior” to the Coronavirus Pandemic and “Currently”; Number of hours seated by day “Prior” to the Coronavirus Pandemic and “Currently”; Participants emotional feelings were categorised into Soothed/Anger (VAS Soothed/Anger) Prior to the Coronavirus Pandemic and Currently, Sadness/Joy (VAS Sadness/Joy) “Prior” to the Coronavirus Pandemic and “Currently”, Peaceful/Excited (VAS Peaceful/Excited) “Prior” to the Coronavirus Pandemic and “Currently”, Busy/Boredom (VAS Busy/Boredom) “Prior” to the Coronavirus Pandemic and “Currently”. To holistically evaluate the three components an additional outcome measure was incorporated to ascertain how the participants felt about the passage of time. The VAS scale was used as a measure to assess the perception of time from the participants’ perspective. The scale of measurement ranged from “time seems to go slowly” to “time flies very fast”, and this was applied “Prior” to the Coronavirus Pandemic, “Currently”, “During the day” and “During the week”. 

### 2.3. Statistical Analysis 

Statistical analysis software IBM SPSS Statistics for Windows, Version 25.0. (IBM Corp., Armonk, NY, USA) was used to analyse the datasets. A Shapiro–Wilks test was used to ascertain whether the data was normally distributed. Descriptive statistics were provided, and a mixed methods approach was applied to the dataset to extract and assess emotional feeling outputs based on countries, gender, age and occupation. The “Prior” and “Currently” sets of score comparisons and associations within the three components, namely, leisure physical activity, sedentary action and emotions were performed using the Wilcoxon signed-rank test and Spearman correlation (r). A Spearman’s correlation (r) between age and all the variables was also calculated. An association between the physical variables (leisure physical activity, sedentary action) and emotion was performed using the Spearman correlation (r) for the “Prior” variables and “Currently” variables, respectively. When implementing the Spearman’s correlation, the r values obtained varied between −1 and +1 where 1 is a perfect correlation and 0 represents no correlation [49,50]. Further interpretations of r include 1 > r ≥ 0.8 (Very Strong); 0.8 > r ≥ 0.6 (Moderate); 0.6 > r ≥ 0.3 (Fair) and 0.3 > r ≥ 0.1 (Poor) [1,2]. *p* < 0.05 was considered significant.

## 3. Results

The Shapiro–Wilks Test revealed a statistically significant difference (*p* < 0.001) indicating that the data was not normally distributed. As a result, we rejected the null hypothesis for a normal distribution. Out of a total of 10,121 participants that completed the COVISTRESS questionnaire, only 952 participants omitted recording their country of domicile, and 27 did not record their sex. A total of 9142 (6292 female (aged: 41.40 ± 13.10 years) and 2850 male (aged: 43.92 ± 14.30 years)) participants that fully completed the COVISTRESS questionnaire were recruited from 67 countries located on the following continents Africa, Asia, Australia, Europe, North America and South America.

Both males and females displayed similar output measures. Table 1 provides a display of the combined sex output measures. Males (“Prior” COVID-19: 13.09 ± 6.40; “Currently”: 15.80 ± 7.70) produced a higher VAS scale in comparison to the Females (“Prior” COVID-19: 12.15 ± 5.95; “Currently”: 15.25 ± 7.55) for the sedentary measurement, i.e., number of hours seated per day. A stronger correlation between “Prior” COVID-19 and “Currently” was also observed among the Males (r = 0.720) in comparison to the Females (r = 0.639). In terms of physical activity measurement, i.e., number of hours of leisure physical activity per week, Males (“Prior” COVID-19: 10.39 ± 8.61; “Currently”: 7.75 ± 7.61) produced higher VAS scale outputs when compared to Females (“Prior” COVID-19: 8.32 ± 7.29; “Currently”: 5.99 ± 6.08). A moderate correlation between “Prior” COVID-19 and “Currently” was observed among the Males (r = 0.541) in comparison to the Females (r = 0.502).

Regarding the emotional feeling measurement, Males produced higher VAS scale outputs in terms of rating their psychological emotions for soothed/anger (“Prior” COVID-19: 37.43 ± 22.28; “Currently”: 49.57 ± 25.74), sadness/joy (“Prior” COVID-19: 68.06 ± 20.35; “Currently”: 51.54 ± 24.34), peaceful/excited (“Prior” COVID-19: 41.22 ± 24.77; “Currently”: 42.72 ± 24.13), busy/boredom (“Prior” COVID-19: 21.18 ± 19.67; “Currently”: 41.00 ± 29.78) than Females soothed/anger (“Prior” COVID-19: 38.44 ± 23.16; “Currently”: 57.15 ± 25.20), sadness/joy (“Prior” COVID-19: 68.78 ± 21.48; “Currently”: 44.97 ± 24.89), peaceful/excited (“Prior” COVID-19: 44.98 ± 25.27; “Currently”: 48.77 ± 25.24), busy/boredom (“Prior” COVID-19: 18.44 ± 19.31; “Currently”: 41.09 ± 31.20). Overall, both males (r < 0.336) and females (r < 0.192) produced a poor correlation between the VAS scale outputs “Prior” COVID-19 and “Currently”. 

The Wilcoxon signed rank test showed significant differences for sedentary activity (Z = −40.462, *p* < 0.001) and physical activity (Z = −30.751, *p* < 0.001) with respect to “Prior” COVID-19 and “Currently”. In terms of the emotional feelings “Prior” and “Currently”, the Wilcoxon signed rank test results were as follows: soothed/anger (Z = −39.970, *p* < 0.001), sadness/joy (Z = −50.217, *p* < 0.001), peaceful/excited (Z = −9.600, *p* < 0.001) and busy/boredom (Z = −47.256, *p* < 0.001), respectively.

**Figure 1:**
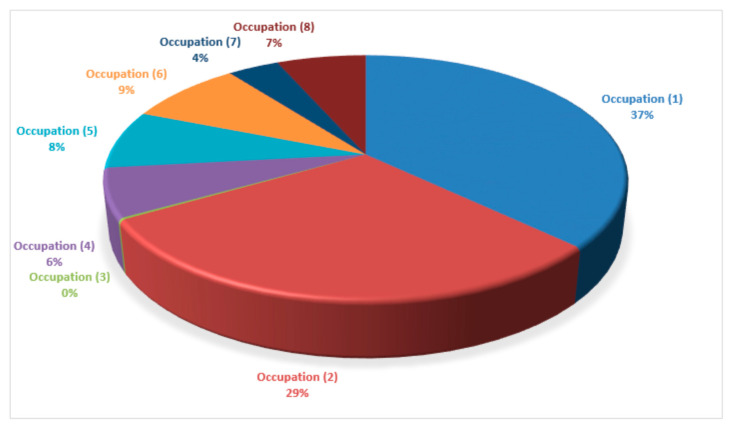
Pie chart display of the total occupation representation from the pool of participants that completed the COVISTRESS questionnaire.

From 10,121 participants that completed the COVISTRESS questionnaire, only 7938 participants provided their occupational status. The following statuses were recorded (number of participant recordings): (1) Executive and superior intellectual occupation (included students in medicine, pharmacy and dentistry) (2944 participants); (2) Intermediary professions, e.g., Teachers, Administrative roles, Clergy, etc. (2342 participants); (3) Farmer (18 participants); (4) Artisan, merchant or entrepreneur (512 participants); (5) Worker (617 participants); (6) Student (677 participants); (7) Looking for a job (303 participants); (8) Retired (525 participants). The percentage participant occupation recordings are displayed in Figure 1.

There were no correlations (r) observed between age and all the measured variables (i.e., −0.001 > r > 0.062). The Wilcoxon signed rank test showed significant differences between the “Prior” Coronavirus Pandemic variable and the “Currently” (Z = −47.604, *p* < 0.001); “During the day” (Z = −43.510, *p* < 0.001); and “During the week” (Z = −41.346, *p* < 0.001) variables. Significant differences were also exhibited between “Prior” and “During the Day” (Z = −19.232, *p* < 0.001); “Currently” and “During the Week” (Z = −22.733, *p* < 0.001) and “During the Day” and “During the Week” (Z = −5.706, *p* < 0.001). 

**Figure 2:**
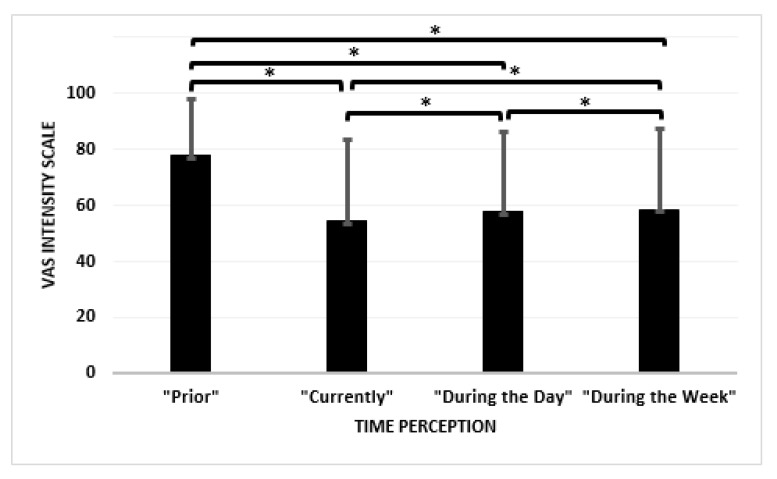
Bar chart description showing the perception of time based on the VAS intensity scale where time perception is measured from a scale from 1 to 100 and interpreted from “time seems to go slowly” to “time flies very fast”. * indicates significance between variables.

There were no correlations (r) between the leisure physical activity and sedentary action (i.e., “Prior” = −0.071; “Currently” = −0.097). Although there were no correlations (r) between leisure physical activity and emotion (i.e., −0.071 > r > 0.081) for “Prior”, the correlations (r) between leisure physical activity and sedentary action (i.e., −0.078 > r > 0.167) for “Current” were poor. The correlations (r) between sedentary action and emotion for “Prior” and “Currently” were (−0.100 > r > 0.075) and (−0.040 > r > 0.041), respectively. Figure 2 provides a bar chart description of how the participants felt about the passage of time. 

Further interpretations of r include: 1 > r ≥ 0.8 (Very Strong); 0.8 > r ≥ 0.6 (Moderate); 0.6 > r ≥ 0.3 (Fair) and 0.3 > r ≥ 0.1 (Poor) [1,2]. *p* < 0.05 was considered significant.

## 4. Discussion

The results presented here provide an insight into the interactions between leisure, physical activity, sedentary action and emotions using the COVISTRESS VAS intensity scale. On completion of the current dataset analyses, a 90% compliance was achieved with respect to completed COVISTRESS questionnaires from participants located in 67 different countries in the following continents Africa, Asia, Australia, Europe, North America and South America. Overall, males produced a higher VAS scale in comparison to the females for all three components, i.e., leisure, physical activity, sedentary action and emotions. Although, the Spearman correlation (*r*) produced no correlation between age and all the measured variables, a moderate correlation was achieved between the “Prior” and “Currently” sets of score comparisons and associations for the sedentary action component; a fair correlation was attained for the leisure physical activity component, and poor correlations were observed for the emotional feelings component. The Wilcoxon signed rank test showed significant differences (*p* < 0.001) between all the perception of time variables, i.e., applied “Prior” to the Coronavirus Pandemic, “Currently”, “During the day” and “During the week”. Among the occupational statuses recorded within the COVISTRESS questionnaire, the executive and superior intellectual occupation (included students in medicine, pharmacy and dentistry) recorded the largest number of participant recordings. This accounted for 2944 participants, which represented 37% from 7938 participants who provided their occupational statuses on the questionnaire. Our results were in agreement with our hypothesis. 

To respond to the evolving unprecedented COVID-19 pandemic outbreak, an online validated self-reported COVISTRESS questionnaire was proposed and administered. This remote option was preferred to interview as using the COVISTRESS questionnaires provided a platform that truly reflected some of the key concerns necessary to understand selective physical and psychological components associated with stress in individual personal life’s and work environment. The concept of using questionnaires also allowed us to broaden our database through the recruitment of participants from sixty-seven countries to date. Whilst the COVISTRESS questionnaire is not a diagnostic “qualitative tool” for COVID-19, it is pertinent that readers appreciate that the outputs reported here are simply a broad assessment highlighting symptoms associated with the COVID-19 situation in response to feedback from a wide variety of citizens scattered across sixty-seven different countries located on six different continents. To date, there has been a rapid growth in the number of publications into the physical health and psychological consequences of COVID-19. A few qualitatively driven studies have used customised COVID-19 related questionnaires to survey the public on issues related to the psychological impact of COVID-19 on mental health. Some of these research studies have emerged from countries such as Australia [51], Spain [52,53,54], India [55,56], Mexico [57], China [58,59,60,61], United Kingdom [62], USA [63], and Italy [64,65]. Based on the completed virtual and web-based surveys, the outcomes from these surveys suggest that levels and rates of psychological distress vary across countries. Spain reported 72% of their survey respondents had elevated psychological distress on the General Health Questionnaire-12 (GHQ-12) [52]. Australia reported that 78% of respondents using their self-reported questionnaire observed that their mental health decreased since the outbreak, with 25.9% reported to have been very or extremely worried about contracting COVID-19, and 52.7% worried about family and friends contracting COVID-19 [51]. India reported that during the COVID-19 pandemic 25%, 28%, and 11.6% exhibited symptoms of moderate to extremely severe depression, anxiety and stress, respectively [66]. Mexico reported 50.3% psychological distress as moderate to severe [57]. China reported in a cross-sectional survey from 52,730 participants conducted between 31 January and 10 February 2020 [61] where 29.3% of the respondents experienced mild to moderate psychological distress, and 5.1% experienced severe distress. In another survey of 1210 members, Wang et al. [58] reported that 53.8% of participants rated the psychological impact of the COVID-19 outbreak as moderate to severe, 75% were worried about their family members contracting COVID-19, and rates of moderate to severe depression, anxiety and stress were 16.5%, 28.8%, and 8.1% respectively. Four weeks later, in a follow-up survey, rates of depression, anxiety and stress remained unchanged [67].

Despite all this information provided via the surveys, there is still limited information on the impact that COVID-19 has had and continues to have on individuals’ providing a powerful reason why more information and research is needed to fully understand the short and long term consequences that the COVID-19 pandemic has had on the mental, physical health and wellbeing of individuals. Our study reported incremental differences in the physical and psychological output measures reported, i.e., “Prior” COVID-19 and “Currently”. These differences are representative indicators of the outcome measures reported, which when interpreted suggest that during COVID-19 (a) most participants adopted a sedentary habit, (b) physical activity per week decreased, (c) more people exhibited anger traits, (d) participants predominantly were sad, (e) participants appeared not at peace with themselves, and (f) a large proportion of participants were bored. In terms of directionality of influences particularly between the physical and emotion variables, our results showed that the associations between the physical variables (sedentary activity and physical activity) and the emotional ones were poor at best. With respect to the emotional feeling measurements “Prior” to COVID-19 and “Currently”, participants increased their sedentary habits by 2.98%, the level of physical activity reduced by 2.42%, depression levels increased by 21.62%, anxiety levels increased by 16.71%, and stress levels increased by 21.8%. These output measures agree with earlier reported levels of depression, anxiety and stress from different countries. These worrying statistics from the reported studies on COVID-19 demonstrate the raised psychological distress in the general community and countries at large. 

This study is a novel study using the COVISTRESS questionnaire, as a limitation our study did not cover quality of life in terms of care, diagnosis, treatment and rehabilitation. Quality assurance was maintained by ensuring only one questionnaire was submitted per IP address. Nevertheless, it was difficult to control, restrict and monitor all participants remotely, i.e., it is possible the same participant could have submitted several questionnaires from different IP addresses. Research by William A. Rushing suggests that marital status and rates of mental health are related [68]. However, the marital status of all participants was not accounted for in this study. Although 10,121 participants that completed the COVISTRESS questionnaires were randomised, the occupational pool of participants was small and could have been larger. This study consisted of a greater proportion of females to males. Unfortunately, gender balance could not be controlled for. Consideration was not given to the fact that different countries/continents experienced COVID-19 at different times, as different rates of evolution and measures to cope with COVID-19 were adopted. Neither countries nor the phase (in terms of confinement measures) were controlled for in this study. 

Future studies need to continue to provide high quality research that explores the impact of COVID-19 globally. This collaborative effort could foster the development of community and healthcare support systems, treatment and prevention efforts and provide informed evidence-based policy decisions that could yield meaningful assistance and preventative measures. These measures may benefit vulnerable individuals in society who are at risk of poor quality of life influenced by physical and psychological health related symptoms associated with the unprecedented and ongoing COVID-19 pandemic.

## 5. Conclusions

The goals of this study were achieved through the effective use and interpretation of the COVISTRESS questionnaire. A detailed assessment and report on the impact of isolation and the effect of coronavirus on selected psychological correlates associated with emotions were presented in this study. The findings indicate that the COVISTRESS project has created awareness in relation to the physical and psychological impact resulting from the COVID-19 pandemic. Although more data is still being collated, the current findings have highlighted individual distress caused by COVID-19 and associated health consequences for the global community.

## Figures and Tables

**Table 1:** Descriptive statistics showing the “Prior” and “Currently” dataset summary of the VAS intensity scale for the three components/output measures.

Output Measures	Assessment “Prior” COVID-19 (Mean ± SD)	Assessment “Currently” during COVID-19 (Mean ± SD)	Mean Difference between “Prior” and “Currently”	Correlation (r) between “Prior” and “Currently”	Statistical Significance
Number of hours seated per day	12.45 ± 6.11	15.43 ± 7.60	2.98	0.656	<0.001
Number of hours of leisure physical activity per week	8.97 ± 7.79	6.55 ± 6.65	−2.42	0.503	<0.001
^a^ Soothed/Anger	38.13 ± 22.90	54.84 ± 25.60	16.71	0.190	<0.001
^b^ Sadness/Joy	68.56 ± 21.14	46.94 ± 24.91	−21.62	0.191	<0.001
Peaceful/Excited	43.86 ± 25.19	46.93 ± 25.06	3.07	0.252	<0.001
^c^ Busy/Bored	19.27 ± 19.46	41.07 ± 30.77	21.8	0.253	<0.001

Note: ^a^ indicates depression, which is represented as Sadness/Joy; ^b^ indicates anxiety, which is represented as Soothed/Anger, and ^c^ indicates stress levels, which are represented as Busy/Bored. Correlation is significant at the 0.01 level (2 tailed).
